# In flux: Associations of substance use with instability in housing, employment, and income among young adults experiencing homelessness

**DOI:** 10.1371/journal.pone.0303439

**Published:** 2024-05-13

**Authors:** Rupa Jose, Elizabeth J. D’Amico, David J. Klein, Anthony Rodriguez, Eric R. Pedersen, Joan S. Tucker

**Affiliations:** 1 RAND Corporation, Arlington, Virginia, United States of America; 2 RAND Corporation, Santa Monica, California, United States of America; 3 RAND Corporation, Boston, Massachusetts, United States of America; 4 Department of Psychiatry and Behavioral Sciences, University of Southern California, Los Angeles, California, United States of America; University of Houston, UNITED STATES

## Abstract

Young adults experiencing homelessness (YAEH) are faced with instabilities in many areas of their lives, including their living situation, employment, and income. Little is known about how the experience of instability in these different domains might be associated with substance use. Leveraging data collected on 276 YAEH in Los Angeles County, regression analyses examine associations between three distinct types of instability (housing, employment, income) and participants’ self-reported alcohol use, alcohol consequences, non-cannabis drug use, and substance use symptoms. Results indicated that recent instability in income, employment, and secure housing for those with access to it (but not housing in general or non-secure housing) were significantly associated with greater alcohol/drug use or substance use symptoms. Depression was also found to moderate the association between employment instability and alcohol use. Our findings suggest that efforts to reduce instability in income, employment, and secure housing may have positive benefits for substance using YAEH, especially those with depressive symptoms.

## Introduction

From January to March 2022, over thirty thousand unaccompanied young people were experiencing homelessness in the United States and most (91%) were between the ages of 18–25 [1]. This age group is in a transition period between adolescence and full adulthood marked by exploration and instability [2]. Substance use importantly tends to peak during this period in the U.S. [3], including among 18–25 year olds experiencing homelessness. Estimates indicate that 13% to 50% of 16–25 year olds experiencing homelessness report an alcohol use disorder and 12% to 60% report substance use disorders more broadly [4]. Prevalence estimates for non-cannabis drug use among 18–26 years olds experiencing homelessness can be between 18% and 8% depending on drug type (e.g., 17.7% cocaine use, 17.5% methamphetamine use, 15.4% ecstasy use, 8.2% crack use, and 8.3% injection drug use) [5]. Combatting homelessness, including among young people, has become a priority for government leaders and researchers given that homelessness is a significant public health problem with increasing social costs [6]. The role of instability in the substance use of young adults experiencing homelessness is likely complex, but understanding it is key to informing efforts to reduce homelessness.

Programs that offer access to needed behavioral health treatments (e.g., substance use disorder treatment [7]) or housing and employment services [8,9] are often touted as important elements in reducing homelessness. The underlying assumption of these programs is that individuals experiencing homelessness fare best when they are provided with *positive stable circumstances*. As homelessness is traditionally defined by shifting states or instability, we investigate whether situational stability across different domains is associated with fewer alcohol and drug use behaviors among young adults experiencing homelessness (YAEH).

Finding a consistent or stable place to live (Housing First [9]), steady employment [8,10], regular income [11], and supportive social connections [12] have all been associated with getting people “off the streets” or helping those formerly unemployed secure employment. The current literature, however, yields mixed results on how stability affects alcohol and drug use behaviors [11,13]. This could be due, in part, to the fact that substance use has often been identified as both a *consequence of homelessness* (i.e., seen at times as a way to cope with life on the streets [14]), as well as a *cause of homelessness* (i.e., alcohol use and drug use disorders increase one’s risk for first-time homelessness by 3.6% and 12%, respectively [15]).

For non-homeless populations, situational stability is associated with mental health and well-being. For example, in a nationally representative study of U.S. adults experiencing housing instability due to the ‘Great Recession’ (2007–2009), adults with more unstable housing circumstances (difficulty paying rent/mortgage or lost homes) reported more alcohol use disorder symptoms and negative drinking consequences than those with stable housing [16]. Work unpredictability (measured by changing tasks, activities, and deadlines) and unemployment have additionally been associated with poor self-reported well-being (percieved evening serenity and strain [17]), along with alcohol and drug use problems (i.e., risky alcohol consumption, alcohol disorders, drug abuse/dependence, and relapse risk following treament [18]). For young adults who are already experiencing the stressor of homelessness, more unpredictability in their daily life would likely be an additional source of stress potentially culminating in alcohol or drug use, yet no studies to date have examined this issue.

### Current study

This study addressed the gap in the current literature by examining associations between recent housing, employment, or income instability (measured over a 3-month or 1-month timeframe prior to study entry) and alcohol use, alcohol consequences, non-cannabis drug use, or general substance use symptoms for YAEH within the Los Angeles area. In the current study, we framed “instability” as the *opposite* of stable, predictable, or consistent circumstances with higher levels of instability defined by more varied housing, employment, or income situations. In a sample experiencing homelessness, employment stability meant consistent unemployment or consistent employment, whereas “instability” meant multiple jobs either part- or full-time. Similarly, income stability in this sample experiencing homelessness was defined as either those who received no money from any named source or those who received income from one benefactor.

Guided by findings in non-homeless populations (16–18), we expected that recent housing, employment, and income instability would be associated with greater self-reported alcohol and drug use. Given the high comorbidity of depression and substance use within the general population [19] and homeless population [20,21], we also examined whether depression moderated the association between different types of instability and self-reported substance use behaviors, adding to the growing body of work aimed at better capturing variability within the homeless experience (e.g., [22]). As prior studies have found that higher depression scores are associated with substance use severity [23], we expected that depression would amplify the association between instability and substance use, such that YAEH with high levels of instability and depression would be the most vulnerable to poor substance use outcomes. To date, no studies have examined the multidimensional nature by which instability can affect health behaviors and outcomes of YAEH; thus, the present study adds significantly to the evolving literature addressing potential modifiable factors that may affect both substance use and homelessness.

## Materials and methods

### Sample

Participants were recruited between November 16, 2018 and March 23, 2021 from three drop-in centers that provide a wide range of services to YAEH within Los Angeles County. They were enrolled in a longitudinal study examining the effects of a brief group motivational interviewing-based intervention on substance use and sexual risk behavior (for additional details; see [24]). To be eligible, prospective participants had to be 18–25 years old, show no cognitive impairment, provide contact information, have access to a phone/e-mail for follow-up, commit to being in the study area for the next month, and be English speaking. Of those approached during the recruitment period (*n* = 371), a total of 276 people (74%) met eligibility criteria, provided written consent, and completed the baseline survey. Analyses focus on data collected at baseline. All materials and procedures were approved by the RAND Human Subjects Protection Committee and all participants provided written informed consent.

### Measures

#### Demographics

Participants self-reported their age (in years), sex at birth (male/intersex/other = 0, female = 1), race and ethnicity (which was coded as three binary variables: non-Hispanic Black/African American; non-Hispanic Asian/Pacific Islander/American Indian/Alaska native/other/mixed race; and Hispanic/Latinx; non-Hispanic White was used as a reference group in regression models), sexual orientation (straight/heterosexual = 0, gay/lesbian/bisexual/questioning/asexual = 1), education (high school diploma/GED or beyond = 0, no high school diploma = 1), income in the past 30 days (in dollars), and current employment status (unemployed = 0, employed full-time or part-time = 1).

#### Length of most recent homelessness

We did not require that participants be currently experiencing homelessness to be in the study; however, most individuals utilizing drop-in centers were experiencing homelessness at the time of study entry (89% [25]). For length of most recent homelessness, we asked: “Think about the most recent period of time when you have not had a regular place to stay. How long has that period of time lasted?” To respond, participants indicated a two-digit numeric value and selected the appropriate time unit (days, weeks, months, or years). All responses were recalibrated to days (i.e., weeks were multiplied by 7, months were multiplied by 30, and years were multiplied by 365).

#### Depression

The 8-item Patient Health Questionnaire (PHQ-8) is a valid and reliable diagnostic tool to screen for depression using the cut-point of 10 or above [26]. Reflecting over the last two weeks, participants report, for example, how often they had trouble concentrating, had a poor appetite or overate, or felt down, depressed, or hopeless. Response options ranged from “not at all” = 0 to “nearly every day” = 3. Item values were summed to create a total PHQ-8 score (range 0–24; sample α = 0.93) and then recoded as a binary variable based on the cut-point value of 10 or greater to indicate probable depression.

#### Generic housing instability

Instability in generic housing was assessed using responses from a multi-item question that asked participants how frequently in the past 3 months they had spent the night in the following 10 places: their own house/apartment/room, someone else’s apartment/house that was a regular place for them to stay, someone else’s apartment/house that was a temporary place for them to stay, an emergency shelter, a transitional housing program, outdoors/the street/a park, a car or other private vehicle, an abandoned building, a hotel/motel, or somewhere else. Responses were originally coded along an 8-point response scale ranging from “never” to “every day.” From this information we derived our measure of generic housing instability as: 1 = one place indicated for “every day”, 2 = one place indicated, but for *not* every day (part of the week), 3 = staying 2 places, 4 = staying 3 places, 5 = staying 4 places, 6 = staying 5 places, 7 = staying 6 places, 8 = staying 7 places, and 9 = staying 8 or more places. This final category combined those staying at 8–10 places as few participants endorsed these responses (1.5%-3.0%) and there was no theoretical reason to distinguish between high generic housing instability states. Generic housing instability is the composite of secure and non-secure housing instability measures discussed below.

#### Secure and non-secure housing instability

We delineated generic housing instability into two categories—secure and non-secure housing—given that prior work shows type of housing matters for substance use vulnerability [27]. Support for our secure and non-secure housing groupings comes from the housing type criterion (non-temporary, roommates not required, and housing support/subsidiaries present) used to develop the Housing Security Scale [28]. Secure housing places included one’s own house/apartment/room, access to someone else’s apartment/house regularly, and transitional housing programs. For the secure housing instability measure, among those who reported staying at least one night at a secure housing place in the past 3 months, their stays were coded as follows: 1 = one secure place indicated for “every day”, 2 = one secure place indicated, but for *not* every day (part of the week), 3 = staying 2 secure places, and 4 = staying at all 3 secure housing places. This coding allows us to capture the degree of stability in a participant’s residence in a secure housing establishment, with values ranging from “stable” (1) to “less stable” (2–4).

By contrast, less secure or non-secure housing places included staying at someone else’s apartment/house temporarily, an emergency shelter, outdoors/the street/a park, a car/other private vehicle, an abandoned building, and a hotel/motel. The term “non-secure housing” is used for simplicity though we recognize this term captures a mix of diverse sheltered and non-sheltered places not always called ‘housing.’ For the non-secure housing stability measure, among those who reported staying at least one night at a non-secure housing place in the past 3 months, their stays were coded as follows: 1 = one non-secure place indicated for “every day”, 2 = one non-secure place indicated, but for *not* every day (part of the week), 3 = 2 non-secure places, 4 = 3 non-secure places, 5 = 4 non-secure places, 6 = 5 non-secure places, and 7 = 6 non-secure places. Again, this coding captures the degree of stability in a participant’s residence in a non-secure housing establishment, with values ranging from “stable” (1) to “less stable” (2–7).

In total, 123 YAEH (45% of our sample) endorsed stays at *both* secure and non-secure housing types. This sizeable overlap meant we were underpowered to detect the unique (non-shared) associations of secure housing instability and non-secure housing instability in a single model. We therefore analyzed separate subset models. Secure housing instability models included any YAEH who indicated at least one night in secure housing (*n* = 149) whereas non-secure housing instability models included any YAEH who indicated at least one night in non-secure housing (*n* = 223).

#### Income instability

Instability in income was assessed using responses from a multi-item question asking how much income (money in dollars) participants received in the past 30 days from the following 5 different sources: a full-/part-time job, friends/relatives, government assistance, miscellaneous/risky activities (i.e., panhandling, dealing drugs, exchanging sex, scrapping, exchanging bottles and cans, stealing, or similar activities), or other sources not mentioned. Binary measures for each of 5 income sources were generated such that 1 = money received and 0 = no money received. Binary variables were then summed to create our measure of income instability with those who received no money from any source treated the same as someone with 1 income source (i.e., the most stable condition). Income instability values ranged from 1 to 5 and represented the following: 1 = no income or 1 income source, 2 = 2 income sources, 3 = 3 income sources, 4 = 4 income sources, and 5 = all 5 income sources.

#### Employment instability

Instability in employment was based on two questions, both asked in relation to the past 3 months. The first asked how many different jobs (part-time or full-time) one had held over the last 3 months, and the second asked the longest time spent in any one job in terms of days, weeks, or months. Combining responses from these two questions, we generated our measure of employment instability. Employment instability was coded such that 0 = no jobs or 1 job for the full 90 days, 1 = 1 job for less than 90 days, 2 = 2 jobs, 3 = 3 jobs, 4 = 4 jobs, 5 = 5 jobs, 6 = 6 jobs, and 7 = 7 or more jobs.

All instability measures (i.e., generic housing, secure and non-secure housing, income, and employment instability) were coded such that higher values reflected greater instability.

#### Alcohol use and consequences

Alcohol use was the product of two variables–number of days in the past 30 days one drank and number of drinks one consumed on days they drank–divided by 30. In essence, this variable captured the average number of drinks per day in the past month (value range: 0–22). We used 14 items from the Brief Young Adult Alcohol Consequences Questionnaire (B-YAACQ [29]) to generate a sum of alcohol consequences in the past 30 days (value range: 0–14). Consequences included a mix of somatic (e.g., sick, lacking energy), psychological (e.g., low self-esteem), and situational (e.g., regrettable sexual encounters or taking foolish risks) adversities resulting from alcohol use (sample α = 0.92).

#### Drug us

Participants reported drug use in the past 30 days (excluding cannabis). We focused on non-cannabis drug use as cannabis is used widely by young people experiencing homelessness [30], including those enrolled in our study (i.e., 78% of participants used cannabis in the past 30 days). For drug use, the original survey item asked number of days the participants used each of the listed 12 different drug types to get high: crack, cocaine, heroin, methamphetamine, ecstasy, hallucinogens, inhalants, over-the-counter medicines, prescription amphetamine medications, prescription sedative medications, prescription tranquilizers, and prescription narcotics. Since over two-thirds of our sample did not use any of the listed drugs (68%), we generated a simplified any drug use measure with 1 = 1 or more non-cannabis drugs used and 0 = no non-cannabis drugs used.

#### Substance use symptoms

The 5-item Global Appraisal of Individual Needs–Short Screener (GAIN-SS [31]) measured substance use symptoms. Participants indicated, for example, the last time that they used alcohol or other drugs weekly, spent a lot of time getting, using, or feeling the effects of alcohol/drugs, or kept using alcohol/drugs even as problems arose. Response options included “never,” “in the past month,” “2–12 months ago” and “1 or more years ago.” Due to the timing of our instability measures (past month or 3 months), a count of substance use symptoms within the past month was generated by adding all “in the past month” responses (count range: 0–5).

### Analyses

Sample descriptives are reported as means (*M*) with standard deviations (*SD*), medians with interquartile ranges (IQR), or percentages. Linear or negative binomial regressions were estimated for most substance use outcomes (alcohol use, alcohol consequences, and substance use symptoms), excluding non-cannabis drug use which was modelled with logistic regressions. For all predictors, coefficient estimates (either unstandardized coefficients, incidence rate ratios, or odds ratio values) as well as their corresponding confidence interval and significance were tabled. To determine the extent that depression moderated the association between the three primary instability types (generic housing instability, income instability, employment instability) and substance use outcomes, interaction terms were included as regression predictors. Only significant interactions were plotted and tabled. To understand substance using vulnerability from secure and non-secure housing instability, separate subset models were also estimated. Secure housing models included any individual who endorsed or at least one night in secure housing, and non-secure housing models included any individual who endorsed at least one night in non-secure housing.

Generated income and employment instability variables combine participants who indicated having “no income” or “no job” with participants who reported having a single source of income or only one job for the entire 3-month period, respectively. Even though such participants experience qualitatively different income and employment circumstances, they were grouped together to reflect stable circumstances. To ensure coding was appropriate, we examined whether those in “stable” income and employment situations (0 = no income vs. 1 = 1 income source and 0 = no employment vs. 1 = 1 job for 3 months) differed on outcomes using the Wilcoxon rank sum test and chi-square or Fisher’s exact tests. We did not find any significant differences, supporting our coding decision for these two instability domains (all *p*-values > 0.05).

## Results

Of the 276 individuals who participated in our survey (see Table 1), 28% identified as female; 30% identified as Hispanic, and 37% identified as non-Hispanic Black or African American. Close to half (43%) identified as a sexual or gender minority, and almost one-third (30%) met the threshold for depression (moderate or severe). On average, participants were 22 years old (*SD* = 1.8; age range: 18–25), reported that their most recent homeless period spanned 518 days (*SD* = 630), and had a past month median income of $363.50; 43% also said they were under 18 years old the first time they left home on their own. Despite their early and or recent housing struggles, close to one-quarter (23%) had a full- or part-time job, 43% had earned their GED or high school diploma, and 25% had attended college or held a college degree or certificate.

**Table 1:** Descriptive statistics (*N* = 276).

	*M*(*SD*)	Median (IQR)	%
Age (in years)	22.08 (1.84)	22 (21;24)	
Sex at birth			
Male/intersex/other			72.46
Female			27.54
Race/Ethnicity			
Non-Hispanic White			16.36
Non-Hispanic Black or African American			36.73
Non-Hispanic other race			17.09
Hispanic or Latinx			29.82
Sexual orientation			
Straight or heterosexual			57.35
Gay/lesbian/bisexual/questioning /asexual			42.65
Educational attainment			
No high school degree			32.12
High school diploma, GED, or beyond^b^			67.88
Income in past month (in dollars)	730.55 (1069.19)	363.5 (192;856.5)	
Employment status			
Employed (full-time/part-time)			22.63
Unemployed			77.37
Depression (PHQ-8 values)	7.07 (6.92)		
Depressed (> = 10)			30.07
Homeless days	518.09 (630.08)	210 (60;730)	
Housing instability (generic)	4.24 (2.51)		
Secure housing instability^c^	2.30 (0.96)		
Non-secure housing instability^d^	3.52 (1.78)		
Income instability	1.84 (0.93)	2 (1;2)	
Employment instability	0.96 (1.65)	0 (0;1)	
Count of alcohol consequences (past month)	3.53 (4.35)	2 (0;6)	
Alcohol use (number of drinks per day–past month)	1.04 (2.60)	0.1 (0.0; 0.8)	
Any (non-cannabis) drug use (past month)			32.25
Substance use symptoms (count; past month)	1.22 (1.56)	1 (0;2)	

***Note*.** Means (*M*), standard deviations (*SD*), and percentages (%) are based on all available (non-missing) data. Additionally, for non-normally distributed continuous predictor or outcome variables tested using the Shapiro-Wilk *W* test for normality (*p* < 0.05), median and interquartile range (IQR) values are also displayed. Some missingness was noted for our measures of race/ethnicity (*n* = 1), sexual orientation (*n* = 4), education, employment, and alcohol use (*n* = 2), employment instability and homeless days (*n* = 3) and housing instability (*n* = 14).

**a Other race group includes those with the following racial designations:** Asian, Pacific Islander, American Indian, Alaskan native, other race, and mixed race.

**b “Beyond” high school or GED included those who indicated earning a vocational/technical degree or certificate, attending college, and earning a college degree**.

c Statistic based on those with at least one night in secure housing (n = 149).

D Statistic based on those with at least one night in non-secure housing (n = 223).

Generic housing instability was common among participants. Only about 1 in 4 (24%) of our sample reported having one place where they stayed nightly over a 3-month period, with the average participant reporting finding shelter across 4 different places. Almost half of participants (45%) reported a stable income situation the month prior, with 15% reporting no income and 30% reporting one income source. Participants reported an average of 2 sources of income (*M* = 1.8; *SD* = 0.9), most of which came from government assistance programs or subsidies (60%), full- or part-time jobs (35%), and friends or relatives (33%). Employment circumstances appeared the most stable with 50% of participants reporting that they were consistently without a job. Combining those stably employed in a single job (*n* = 27) with those who were stably unemployed (*n* = 137), 60% of our sample reported “employment stability” over a 3-month period.

Correlations of our three primary instability measures (generic housing, income, and employment instability) indicated only a small positive association across instability types (*r* = 0.1–0.2), suggesting these are related but distinct concepts. Correlations of the three housing instability measures (generic, secure, and non-secure) indicate that secure housing (defined by stays in one’s own home/apartment/room, another’s home/apartment for which one enjoys regularly, or transitional housing) instability was moderately correlated to non-secure housing (defined by stays in cars, emergency shelters, abandoned buildings etc.) instability (*r* = 0.3), and generic housing instability was highly correlated with both secure housing instability (*r* = 0.5) and non-secure housing instability (*r* = 0.9). The noted modest or high correlations are due to the fact that generic housing instability is the combination of secure and non-secure housing stays, and secure/non-secure housing instability measures are not mutually exclusive (with 45% of people reporting both).

In terms of recent (i.e., the past 30 days henceforth referred to as “past month”) substance use, on average participants reported having 1 drink per day (*SD* = 2.6) with around 10% of individuals reporting that they consumed an average of 3 or more drinks per day. Participants self-reported experiencing 3 to 4 alcohol-related consequences over the past month (*SD* = 4.4). About one third (32%) reported non-cannabis drug use in the past month. The mean substance use symptom score for the sample was 1.2 (*SD* = 1.6), which falls into the “moderate” score range (1–2). Moderate substance use symptom scores suggest the possibility of a substance use problem and the need for additional assessments [32].

### Correlates of recent alcohol use or alcohol consequences

#### Recent alcohol use

The average number of drinks per day in the past month was associated with employment instability (*p* = 0.003), but not with generic housing or income instability (see Table 2 “Alcohol Use” panel under “A. Full Sample”). For each unit increase in employment instability, individuals reported 0.24 additional drinks per day in the past month, suggesting that having multiple jobs was associated with heavier drinking behavior compared to being stably employed in a single job or stably unemployed over a three-month period. Individuals who met the clinical threshold for moderate or severe depression reported a greater number of drinks than those with low or no depression by 0.53 drinks. Number of days homeless during the most recent homeless period was also associated with a larger number of drinks, although this association was small (i.e., average number of drinks per day in the past month only increased by 0.04 drinks with each additional 100 days experiencing homelessness).

**Table 2:** Regression results for alcohol consumption, alcohol consequences, general substance use symptoms, and any drug use.

	Alcohol Use^a^^,^^b^		Alcohol Consequences		Substance Use Symptoms		Any Drug Use	
Full Sample	*b*	95% *CI*	*IRR*	95% *CI*	*IRR*	95% *CI*	*OR*	95% *CI*
Age (in years)	0.01	(-0.12, 0.15)	0.98	(0.86, 1.11)	1.07	(0.97, 1.18)	1.13	(0.95, 1.35)
Female	0.00	(-0.53, 0.54)	0.92	(0.56, 1.51)	0.64*	(0.42, 0.96)	1.22	(0.62, 2.41)
Non-Hispanic Black	0.38	(-0.30, 1.05)	1.95*	(1.05, 3.61)	1.68†	(1.00, 2.84)	1.61	(0.64, 4.06)
Non-Hispanic Other race	0.10	(-0.69, 0.88)	1.31	(0.65, 2.65)	0.97	(0.52, 1.80)	1.17	(0.41, 3.37)
Hispanic	0.55	(-0.14, 1.24)	2.56**	(1.34, 4.88)	1.89*	(1.12, 3.18)	3.04*	(1.20, 7.67)
LGBQA^c^	-0.44†	(-0.92, 0.04)	0.71	(0.45, 1.13)	0.81	(0.56, 1.15)	0.86	(0.46, 1.59)
Less than high school	0.08	(-0.46, 0.61)	0.94	(0.59, 1.50)	1.08	(0.74, 1.58)	1.48	(0.74, 2.96)
Income	-0.00	(-0.00, 0.00)	1.00	(1.00, 1.00)	1.00	(1.00, 1.00)	1.00	(1.00, 1.00)
Employed	-0.38	(-0.95, 0.20)	0.74	(0.45, 1.22)	0.73	(0.48, 1.11)	0.51†	(0.23, 1.12)
Depressed status	0.53*	(0.02, 1.03)	1.88**	(1.22, 2.89)	1.69**	(1.20, 2.38)	1.61	(0.87, 2.99)
Homeless days	0.00*	(0.00, 0.00)	1.00	(1.00, 1.00)	1.00	(1.00, 1.00)	1.00	(1.00, 1.00)
*General housing instability*	0.06	(-0.03, 0.15)	1.04	(0.96, 1.13)	1.05	(0.99, 1.12)	1.06	(0.95, 1.20)
*Income instability*	-0.03	(-0.30, 0.24)	1.21	(0.96, 1.53)	1.20†	(1.00, 1.45)	1.92***	(1.34, 2.75)
*Employment instability*	0.24**	(0.08, 0.39)	1.00	(0.87, 1.16)	1.00	(0.91, 1.11)	0.78*	(0.62, 0.99)
Constant	-0.25	(-3.42, 2.92)	1.57	(0.10, 25.55)	0.11†	(0.01, 1.04)	0.00**	(0.00, 0.25)
*F* (*df*, *df*) or LR χ^2^ (*df*)^d^	2.07* (14, 233)		23.79* (14)		38.05*** (14)		34.27** (14)	
*R*^*2*^ or Pseudo *R*^*2*^	0.1106		0.0207		0.0495		0.1080	
*N* observations	248		251		251		251	
B. Secure Housed Subset	*b*	95% *CI*	*IRR*	95% *CI*	*IRR*	95% *CI*	*OR*	95% *CI*
Age (in years)	-0.04	(-0.20, 0.13)	0.97	(0.83, 1.13)	1.04	(0.92, 1.17)	1.24†	(0.96, 1.61)
Female	-0.13	(-0.76, 0.49)	0.72	(0.38, 1.35)	0.52**	(0.32, 0.85)	0.83	(0.33, 2.11)
Non-Hispanic Black	0.48	(-0.35, 1.30)	2.25†	(0.99, 5.11)	1.59	(0.82, 3.10)	1.40	(0.37, 5.34)
Non-Hispanic Other race	-0.07	(-1.10, 0.95)	1.49	(0.56, 3.98)	1.50	(0.69, 3.25)	0.77	(0.15, 3.91)
Hispanic	0.25	(-0.63, 1.14)	2.40*	(1.02, 5.62)	1.57	(0.80, 3.09)	2.97	(0.76, 11.66)
LGBQA^c^	-0.16	(-0.73, 0.42)	0.97	(0.56, 1.69)	1.06	(0.71, 1.60)	1.50	(0.65, 3.47)
Less than high school	-0.25	(-0.89, 0.39)	0.61	(0.33, 1.12)	0.94	(0.61, 1.44)	1.12	(0.43, 2.90)
Income	-0.00	(-0.00, 0.00)	1.00	(1.00, 1.00)	1.00	(1.00, 1.00)	1.00	(1.00, 1.00)
Employed	-0.47	(-1.13, 0.18)	0.48*	(0.26, 0.87)	0.66†	(0.42, 1.03)	0.36†	(0.13, 1.03)
Depressed status	0.50	(-0.10, 1.11)	1.81*	(1.03, 3.17)	1.37	(0.92, 2.05)	1.24	(0.53, 2.93)
Homeless days	0.00†	(-0.00, 0.00)	1.00	(1.00, 1.00)	1.00	(1.00, 1.00)	1.00	(1.00, 1.00)
*Secure housing instability*	0.39*	(0.09, 0.69)	1.41*	(1.06, 1.87)	1.20†	(0.97, 1.48)	1.17	(0.75, 1.82)
*Income instability*	0.08	(-0.23, 0.38)	1.22	(0.93, 1.61)	1.24*	(1.02, 1.51)	2.38**	(1.46, 3.88)
*Employment instability*	0.23*	(0.05, 0.41)	1.01	(0.85, 1.21)	0.96	(0.85, 1.08)	0.81	(0.60, 1.10)
Constant	0.04	(-3.76, 3.84)	1.16	(0.03, 38.75)	0.15	(0.01, 2.21)	0.00*	(0.00, 0.18)
*F* (*df*, *df*) or LR χ^2^ (*df*)^d^	1.87* (14, 127)		23.43† (14)		30.02** (14)		27.78* (14)	
*R*^*2*^ or Pseudo *R*^*2*^	0.1707		0.0343		0.0659		0.1533	
*N* observations	142		145		145		145	
C. Non-Secure Housed Subset	*b*	95% *CI*	*IRR*	95% *CI*	*IRR*	95% *CI*	*OR*	95% *CI*
Age (in years)	0.04	(-0.12, 0.20)	1.01	(0.88, 1.15)	1.07	(0.96, 1.19)	1.14	(0.94, 1.38)
Female	0.20	(-0.44, 0.85)	1.07	(0.62, 1.85)	0.63*	(0.40, 1.00)	1.25	(0.57, 2.71)
Non-Hispanic Black	0.53	(-0.24, 1.30)	2.34**	(1.23, 4.43)	1.85*	(1.06, 3.22)	1.61	(0.60, 4.28)
Non-Hispanic Other race	0.20	(-0.66, 1.06)	1.53	(0.75, 3.11)	1.04	(0.55, 1.94)	1.09	(0.37, 3.22)
Hispanic	0.72†	(-0.05, 1.49)	2.69**	(1.39, 5.18)	1.92*	(1.12, 3.30)	2.88*	(1.09, 7.59)
LGBQA^c^	-0.54†	(-1.10, 0.02)	0.69	(0.42, 1.12)	0.79	(0.54, 1.15)	0.67	(0.34, 1.33)
Less than high school	0.21	(-0.42, 0.84)	1.08	(0.65, 1.79)	1.20	(0.80, 1.80)	1.60	(0.74, 3.46)
Income	-0.00	(-0.00, 0.00)	1.00	(1.00, 1.00)	1.00	(1.00, 1.00)	1.00	(1.00, 1.00)
Employed	-0.28	(-0.95, 0.38)	0.72	(0.42, 1.22)	0.81	(0.52, 1.25)	0.54	(0.23, 1.23)
Depressed status	0.61*	(0.05, 1.17)	2.00**	(1.29, 3.10)	1.74**	(1.22, 2.48)	1.66	(0.86, 3.21)
Homeless days	0.00†	(-0.00, 0.00)	1.00	(1.00, 1.00)	1.00	(1.00, 1.00)	1.00	(1.00, 1.00)
*Non-secure housing instability*	0.01	(-0.14, 0.15)	1.03	(0.91, 1.15)	1.03	(0.94, 1.14)	1.10	(0.92, 1.31)
*Income instability*	-0.13	(-0.44, 0.18)	1.20	(0.94, 1.53)	1.20†	(0.98, 1.46)	1.77**	(1.20, 2.59)
*Employment instability*	0.32**	(0.13, 0.51)	1.02	(0.88, 1.19)	1.00	(0.89, 1.12)	0.79†	(0.61, 1.01)
Constant	-0.56	(-4.19, 3.08)	0.87	(0.04, 17.82)	0.11†	(0.01, 1.19)	0.00*	(0.00, 0.36)
*F* (*df*, *df*) or LR χ^2^ (*df*)^d^	2.25** (14, 197)		22.73† (14)		33.63** (14)		27.05* (14)	
*R*^*2*^ or Pseudo *R*^*2*^	0.1379		0.0228		0.0499		0.0989	
*N* observations	212		214		214		214	

a Analyses excluded two extreme outcome values equal or greater than 20 drinks per day in the past month.

**b Alcohol use models when estimated with robust *SE* (as opposed to the tabled regular *SE*), resulted in similar conclusions for our key instability measures with the only significant shifts noted for employment instability.** Namely, including robust *SE* meant associations between employment instability and alcohol use were either significant at *p* <0.1 (full and non-secure housed samples) or non-significant (secure housed sample).

c LGBQA = Lesbian, Gay, Bisexual, Questioning, and Asexual.

**d Linear regression model fit was indicated by *F* and *R*-squared values for alcohol use whereas negative binomial and logistic regression model fit was indicated by LR χ**^**2**^ **and Pseudo *R*-squared values for alcohol consequences, substance use symptoms, and any drug use.**

† p < 0.1.

*p < 0.05.

**p < 0.01.

***p < 0.001.

#### Secure and non-secure subsamples: Recent alcohol use

Restricting results to only those participants who stayed at least one night in non-secure housing (*n* = 223), we found significant associations of employment instability and depression with past month alcohol use or average number of drinks (see Table 2 “Alcohol Use” panel under “C. Non-Secure Housed Subset”). When restricting to those participants who stayed at least one night in secure housing (*n* = 149), both secure housing instability and employment instability were significantly associated with recent alcoholic drink averages (see Table 2 “Alcohol Use” panel under “B. Secure Housed Subset”). As participants’ stays in secure housing became more varied over a 3-month period (i.e., time spent in only one secure housing place decreased or the number of reported secure housing places stayed increased, for those with access to secure housing), the average number of alcoholic drinks drank per day in the past month increased by an additional 0.39 drinks. The effect size of secure housing instability on alcohol use (β = 0.22) was comparable to the effect size of employment instability on alcohol use (β = 0.23).

#### Recent alcohol consequences

Self-reported alcohol consequences in the past month were not significantly associated with any instability variables when estimating for the full sample (see Table 2 “Alcohol Consequences” panel under “A. Full Sample”). However, identifying as Hispanic or non-Hispanic Black/African American, or screening positive for depression were each associated with reporting around two times the rate of alcohol consequences when compared to identifying as non-Hispanic White or being non-depressed, in the full sample.

#### Secure and non-secure subsamples: Recent alcohol consequences

For those securely housed, becoming less able to rely on regular secure housing (i.e., instability increased) was associated with a greater number of self-reported alcohol consequences, with each incremental increase in secure housing instability corresponding to a 41% increase in the rate of alcohol consequences. Meeting the threshold for depression was again associated with around two times the rate of alcohol consequences when compared to non-depressed persons with access to secure housing. For only those who were securely housed, we found that being employed was associated with a 52% decrease in the rate of alcohol consequences when compared to not being employed (see Table 2 “Alcohol Consequences” panel under “B. Secure Housed Subset”). For those non-securely housed, only minority racial/ethnic identity (Hispanic or non-Hispanic Black/African American status) or screening positive for depression were associated with a greater number of self-reported alcohol consequences (see Table 2 “Alcohol Consequences” panel under “C. Non-Secure Housed Subset”).

### Correlates of recent drug use or substance use symptoms

#### Recent drug use

For the full sample, any non-cannabis drug use in the past month was associated with income instability, employment instability, and reporting Hispanic identity (see Table 2 “Any Drug Use” panel under “A. Full Sample”). For each unit increase in income instability (the addition of another income source), the odds of using non-cannabis drugs increased by 92%. Employment instability had the opposite finding such that for each unit increase in employment instability, the odds of reporting recent non-cannabis drug use decreased by 22%. Moreover, the odds of reporting any non-cannabis drug use were over 3 times higher for individuals who identified as Hispanic as opposed to non-Hispanic White individuals.

#### Secure and non-secure subsamples: Recent drug use

Among securely housed participants, only income instability was significantly associated with an increased odds of non-cannabis drug use in the past month (138% increase in non-cannabis drug use per unit increase in income instability; see Table 2 “Any Drug Use” panel under “B. Secure Housed Subset”). Among non-securely housed participants, Hispanic identity (*OR* = 2.88) along with income instability (77% increase in non-cannabis drug use per unit increase in income instability) were associated with an increased odds of non-cannabis drug use in the past month (see Table 2 “Any Drug Use” panel under C. Non-Secure Housed Subset”).

#### Substance use symptoms

Past month GAIN-SS substance use symptoms in the full sample were correlated with some of the same variables in previous models–depressed state, Hispanic identity–along with sex at birth (see Table 2 “Substance Use Symptoms” panel under “A. Full Sample”). Depressed or Hispanic individuals reported more substance use symptoms (an increase of 69%-89%) than non-depressed or non-Hispanic White persons, respectively. Female sex, however, was negatively associated with substance use symptoms, with females reporting a lower rate of substance use symptoms when compared to non-female (male or other sex) individuals.

#### Secure and non-secure subsamples: Substance use symptoms

Our model of securely housed participants indicated that in addition to non-female identity, income instability was significantly associated with a higher rate of GAIN-SS substance use symptoms (see Table 2 “Substance Use Symptoms” panel under “B. Secure Housed Subset”). Specifically, a one-unit increase in income instability, or the addition of another income source, was associated with a 24% increase in reported substance use symptoms among those who had access to secure housing. Among non-securely housed participants, identifying as depressed, non-Hispanic Black, or Hispanic, were each associated with reporting around twice the rate of substance use symptoms when compared to non-depressed or non-Hispanic White persons (see Table 2 “Substance Use Symptoms” panel under “C. Non-Secure Housed Subset”).

### Testing depression as a moderator

**Fig 1:**
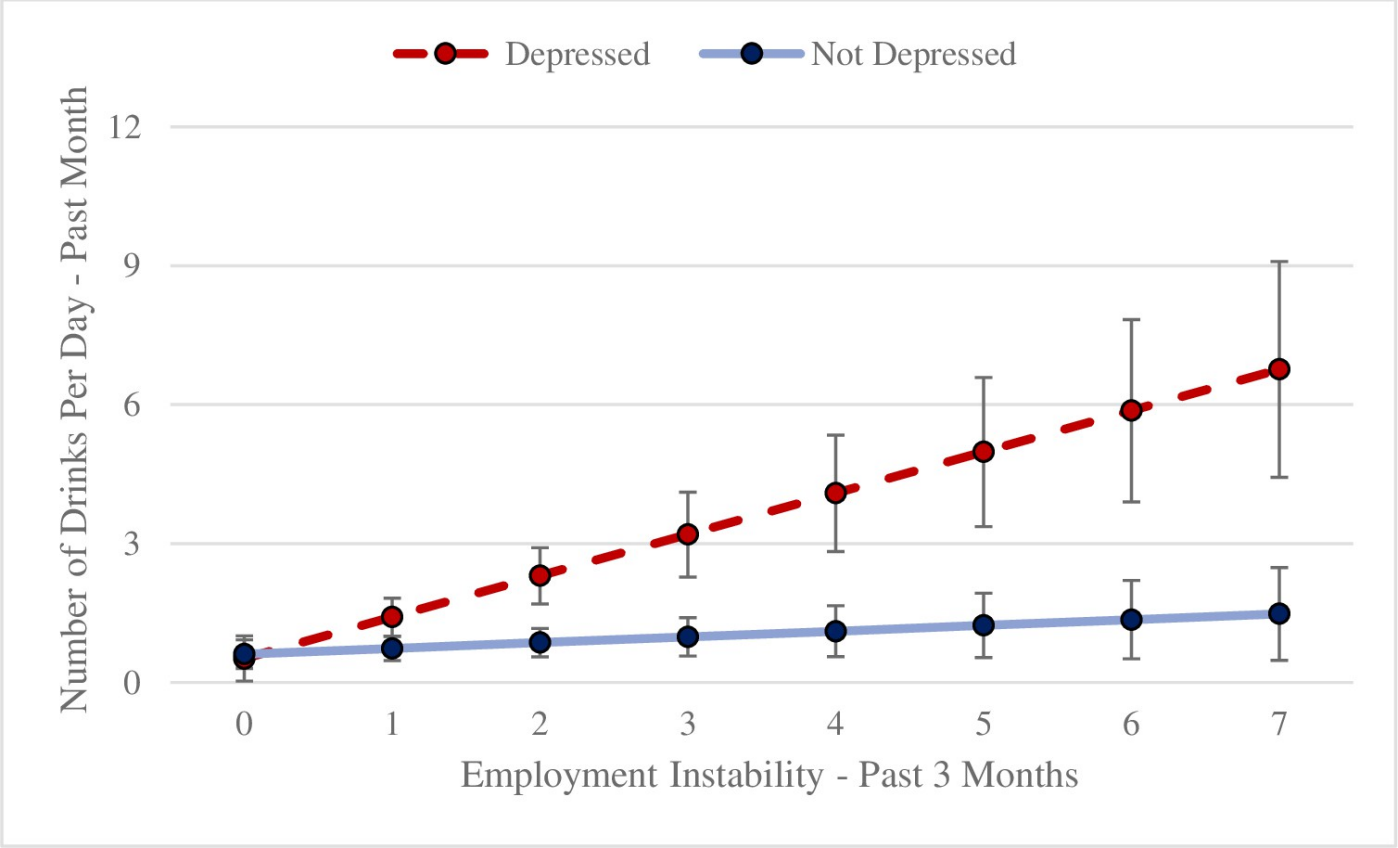
Drinking rates based on depressed status and employment instability. This figure plots depressed status measured of the past two weeks using the PHQ-8 (threshold value of 10) and employment instability measured in the past 3 months to show recent (past month) self-reported alcohol consumption.

In full sample models where at least one form of instability was found to have a significant main effect, we estimated an interaction term with depression (for complete details see Table 3 and Fig 1). The only significant interaction term was between employment instability and depression in our alcohol use model. At the lowest level of employment instability (0 = no job or employed 90 days in one job) the average number of drinks per day was not different for those depressed versus non-depressed. Yet, when employment instability increased, notable differences emerged whereby depressed individuals with greater employment instability reported consuming more drinks per day in the past month than non-depressed individuals (*b* = 0.77; *SE* = 0.20; *p* < 0.001), controlling for relevant covariates.

**Table 3:** Interacting employment instability and depressed status in alcohol drinking rate model.

	*b*	95% *CI*	*p*
Age (in years)	-0.01	(-0.14, 0.13)	0.925
Female	0.01	(-0.51, 0.52)	0.984
Non-Hispanic Black	0.35	(-0.31, 1.01)	0.294
Non-Hispanic Other race	0.17	(-0.60, 0.93)	0.662
Hispanic	0.51	(-0.16, 1.18)	0.132
LGBQA^a^	-0.47	(-0.94, -0.00)	0.048
Less than high school	0.06	(-0.45, 0.58)	0.808
Income	0.00	(-0.00, 0.00)	0.799
Employed	-0.58	(-1.14, -0.01)	0.047
Depressed status	-0.09	(-0.68, 0.49)	0.752
Homeless days	0.00	(0.00, 0.00)	0.022
Housing instability	0.04	(-0.05, 0.13)	0.368
Income instability	-0.01	(-0.27, 0.26)	0.951
Employment instability	0.12	(-0.04, 0.29)	0.131
Employment instability x Depressed	0.77	(0.37, 1.16)	0.000
Constant	0.34	(-2.75, 3.44)	0.827
*F* (*df*, *df*)	3.03 (15, 232)		0.000
*R*-squared	0.1636		
*N* observations	248		

Note. The interaction between employment instability and depression remains significant a p < 0.1 when modeled using a linear regression with robust SE.

a LGBQA = Lesbian, Gay, Bisexual, Questioning, and Asexual.

## Discussion

Instability often permeates the lives of YAEH, traversing multiple domains including where they rest, work, and generate income. Efforts to measure or understand instability for homeless populations typically focus on one’s housing circumstances [28], ignoring other forms of instability thought to drive and maintain homelessness (e.g., income and employment [12]). We thus measured instability in multiple forms to understand the association between domain-specific instability and self-reported alcohol and drug use among YAEH living within Los Angeles County. In addition, we conceptualized instability as a non-consistent, singular state given our focus on a population of YAEH who often experience great inconsistency in their lives.

Full sample models indicated that two forms of instability, employment and income instability, were key in understanding the alcohol and drug use behaviors of YAEH. An increase in employment instability (characterized by multiple jobs) was associated with an increase in average number of reported drinks per day in the past month, and an increase in income instability (characterized by multiple sources of income) was associated with higher rates of non-cannabis drug use. Generic housing instability (characterized by the number of housing places reported and/or time spent in housing), however, was not associated with any outcomes. Person-level demographics (gender, race, ethnicity), homeless duration, and depression were each also found to be significantly associated with substance use, especially alcohol use and consequences. Consistent with existing literature, we found that people of color were more likely to report negative alcohol consequences [33] and drug use [34] than non-Hispanic Whites.

### Having less regular “secure” housing is what matters

YAEH frequently indicate fluctuations in housing stability [35]. Homelessness for this reason often operates more along a “spectrum” than exists as a discrete state. It is in fact common for one to move from a life on the street to a relative’s or friend’s couch [36] and back (a few times) before being able to fully re-transition to stable housing. For this reason, we created variables that captured generic housing instability (irrespective of housing type) as well as instability specific to secure (own home/apartment/room, etc.) and non-secure (car, hotel/motel, outdoors, etc.) housing stays. Secure and non-secure housing instabilities were evaluated in subsample models.

Results from these subsample models highlight that greater instability in secure housing (among those with access to secure housing) was significantly associated with more alcohol use and alcohol-related consequences. We did not find significant association for generic housing instability or non-secure housing instability and alcohol use or consequences. The importance of secure housing stays for alcohol behaviors could be related to the place-based comforts and social capital that typically characterize secure housing.

YAEH who have recently experienced the comforts of secure housing (e.g., a safe, physically covered, and often unrestricted space to rest) might find instability of any kind particularly detrimental to their well-being as loss of a secure, stable place can be a difficult and disruptive adjustment. By contrast, YAEH shifting between non-secure housing stays (which offer few comforts) may be less reactive to greater place-based hardships. To stay a night in your own home, have regular access to someone else’s home, or to participate in a transitional housing program signals that YAEH still have mainstream resources at their disposal in the form of relationships, information, or assets. Close peers or partners [37], family members (e.g., parents [38]), and other supportive adults [39] have been found to protect young people against increased substance use. The diminishment of regular secure housing opportunities (i.e., increases in secure housing instability) for young people who are familiar with secure housing might therefore also lead to increased alcohol issues by diminishing their access to protective individuals who would otherwise be personally invested and able (through frequent monitoring and support) to help prevent them from harmful substance use.

### Income and employment instability are associated with substance use

Having more financiers (more income instability) or employers (more employment instability) in one’s life was associated with greater alcohol and drug use among YAEH, which could signal coping with life event stress by getting drunk or high. High employment instability, for example, arises when an individual is frequently fired or concurrently juggling multiple jobs, both stressful circumstances in which alcohol might be used to cope with job pressures [40,41]. In our population, income instability similarly is believed to arise due to the absence of sufficient resources as 60% received government support. Financial struggles remain a frequent concern of homeless [42] and non-homeless populations, as well as an established risk factor for alcohol and drug use [43].

### Employment instability and alcohol use among depressed YAEH

Young people who reported depression and experienced more employment instability reported heavier past month drinking than young people who did not report depression. A diathesis/vulnerability-stress framework [44] suggests that depression may act as the vulnerability factor in the presence of a stressful situation (high employment instability), resulting in harmful or excessive alcohol use. Finding that depressed individuals might struggle more with inconsistent employment is not entirely surprising and highlights that YAEH with depressive disorders might face additional hurdles when first entering or re-entering the workforce.

## Limitations

The promise of our results come with certain limitations. First, our measures of instability were imperfect. For employment instability, we do not know if individuals held multiple jobs concurrently or sequentially within the 3-month recall period, nor do we know the reasons for multiple prior jobs (e.g., whether they were fired or wanted extra money). Additionally, although we believe that measured instabilities are stressful for our participants, we did not actually assess how “stressful” they found diversity in jobs, income, or places to stay to be and/or the degree they yearned for greater stability. Future studies would benefit from assessing the psychosocial effects of employment, income, and housing instability for YAEH, along with reasons behind such forms of instability and their preferred circumstances. Second, as we were interested in how different types of instability relate to YAEH behavior, we combined different qualitatively distinct forms of stability together (e.g., no job with one regular job). Studies interested in unpacking how employment or income stability “quality” is related to substance use among YAEH should recruit such subgroups at the outset. Third, our survey did not collect granular data on how often participants shifted from a secure to a non-secure housing stay. Such information could be used to better understand frequency of housing shifts and how often people are *shifting between* different housing types as opposed to *shifting within* a given housing type. Relatedly, our housing subset models excluded 13 participants who said they stayed the night “somewhere else” as we did not know if their reply was a secure or non-secure other place. Follow-up work would benefit from replacing such a response with a fill-in item. Due to the considerable overlap of those with secure and non-secure stays and our modest sample of YAEH, we also chose to estimate separate subset models. This modeling choice, while appropriate, prevents us from knowing the effect of secure housing instability adjusting for non-secure housing instability or vice versa. Replication studies with access to large samples should however consider creating mutually exclusive secure and non-secure housing instability measures to see if study findings hold under such conditions. Finally, while we rely on a rich dataset of YEAH, our analyses are cross-sectional in nature. For two of our instability types (employment and housing instability), instability is captured over the previous 3-month period and correlated with substance use outcomes assessed over the prior month. Although these are not longitudinal associations, the temporal sequencing of instability variables and outcomes suggests that a longitudinal association between instability and substance use may in fact exist. Additional analyses with longitudinal data are needed to determine direction of such associations or the true causal pathway. Also, while here we have focused on recent or “acute” forms of instability, associations between chronic forms of instability and short- and long-term substance use remain an important and unaddressed area for YAEH.

## Conclusion

Overall, results suggest that instability across different lifestyle sectors separately, and in a select case in conjunction with depression, are associated with greater substance use and problems. Thus, solutions attending to the situational and psychological correlates of homelessness show promise for ameliorating substance use within YEAH populations. Although our study was not designed to unpack mechanisms by which instability is associated with substance use for those experiencing homelessness, this is as an area ripe for research. For YAEH, being in flux or bouncing between secure places to stay, jobs, or funding sources is associated with greater alcohol and drug use. Researchers and policy makers invested in the welfare of those who experience homelessness are therefore encouraged to invest in programs that cultivate stability (e.g., Housing First [9,45]) or provide in-roads towards stability (e.g., vocation-focused initatives like the Moving Ahead Program [46]).
